# Formation and characteristics of biomimetic mineralo-organic particles in natural surface water

**DOI:** 10.1038/srep28817

**Published:** 2016-06-28

**Authors:** Cheng-Yeu Wu, Jan Martel, Tsui-Yin Wong, David Young, Chien-Chun Liu, Cheng-Wei Lin, John D. Young

**Affiliations:** 1 Laboratory of Nanomaterials, Chang Gung University, Taoyuan, Taiwan, Republic of China; 2 Center for Molecular and Clinical Immunology, Chang Gung University, Taoyuan, Taiwan, Republic of China; 3 Research Center of Bacterial Pathogenesis, Chang Gung University, Taoyuan, Taiwan, Republic of China; 4 Center for General Education, Chang Gung University of Science and Technology, Chiayi, Taiwan, Republic of China; 5 Department of Materials Science and Engineering, Massachusetts Institute of Technology, Cambridge, Massachusetts, United States of America; 6 Graduate Institute of Biomedical Sciences, College of Medicine, Chang Gung University, Taoyuan, Taiwan, Republic of China; 7 Biochemical Engineering Research Center, Ming Chi University of Technology, New Taipei City, Taiwan, Republic of China; 8 Laboratory of Cellular Physiology and Immunology, The Rockefeller University, New York, New York, United States of America

## Abstract

Recent studies have shown that nanoparticles exist in environmental water but the formation, characteristics and fate of such particles remain incompletely understood. We show here that surface water obtained from various sources (ocean, hot springs, and soil) produces mineralo-organic particles that gradually increase in size and number during incubation. Seawater produces mineralo-organic particles following several cycles of filtration and incubation, indicating that this water possesses high particle-seeding potential. Electron microscopy observations reveal round, bacteria-like mineral particles with diameters of 20 to 800 nm, which may coalesce and aggregate to form mineralized biofilm-like structures. Chemical analysis of the particles shows the presence of a wide range of chemical elements that form mixed mineral phases dominated by calcium and iron sulfates, silicon and aluminum oxides, sodium carbonate, and iron sulfide. Proteomic analysis indicates that the particles bind to proteins of bacterial, plant and animal origins. When observed under dark-field microscopy, mineral particles derived from soil-water show biomimetic morphologies, including large, round structures similar to cells undergoing division. These findings have important implications not only for the recognition of biosignatures and fossils of small microorganisms in the environment but also for the geochemical cycling of elements, ions and organic matter in surface water.

Nanomaterials have received widespread attention in recent years due to their possible technological and biomedical applications^1^. Nanoparticles (NPs) not only show enhanced reactivity and penetration in human tissues but they may also have characteristics that differ from the properties of the corresponding bulk material^2^. For these reasons, concerns over the safety and environmental repercussions of these nanomaterials have been the subject of intense debates. Current research initiatives aim to identify the NPs found in the environment and assess the possible effects of such nanomaterials on human health.

While some NPs found in the environment are synthetic or anthropogenic in origin, the major part of such NPs is the results of natural processes^3^,^4^. Natural NPs have indeed been found in the air, soil and water throughout the environment^5^. These NPs and colloids originate mainly from the decomposition of organic matter and chemical weathering of rocks^4^. The main organic chemicals found in surface water particles are humic matter, peptides, proteins, peptidoglycans and polysaccharides while their major mineral components consist of silicates, oxides and hydroxides containing iron, manganese and aluminum^6^. Formation of mineral NPs may produce turbidity and affect drinking water quality, a phenomenon that may cause gastrointestinal illnesses and toxicity if the water is not properly treated or if the particles are found in the water following treatment^7^,^8^,^9^. Yet, our understanding of the formation, characteristics and fate of such particles in surface water environments is incomplete.

We observed earlier that mineral NPs form in body fluids of humans and animals^10^,^11^,^12^,^13^,^14^,^15^,^16^,^17^,^18^,^19^,^20^,^21^,^22^,^23^,^24^,^25^,^26^. These mineral NPs were initially thought to represent nanobacteria, a putative microorganism described as the smallest form of life and the cause of several human diseases, including atherosclerosis, cancer, and kidney stones^27^,^28^,^29^,^30^. On the other hand, our work has shown that these NPs actually represent non-living mineral particles that mimic living microorganisms in various ways, including by their increase in size and number in culture^11^,^17^, their binding to biological molecules (carbohydrates, lipids, metabolites, nucleotides, and proteins)^11^,^12^,^13^,^16^,^20^,^22^,^26^, and by assuming biomimetic morphologies^10^,^18^. The conclusion that nanobacteria represent non-living mineral particles is supported by several studies performed by other authors^31^,^32^,^33^. Notably, the same NPs have been shown to form in the human body^22^,^25^ and may represent precursors of calcification that induce innate immune reactions when they aggregate and form large mineral particles (>1 μm)^19^,^22^. Similarly, Jahnen-Dechent and colleagues have shown that mineral nanoparticles, which this group calls “calciprotein particles”, form in human body fluids^34^,^35^ and that serum proteins such as fetuin-A may play a role in the formation of such particles in the body^36^,^37^.

In the present study, we examine the possibility that biomimetic mineralo-organic NPs may also exist in natural surface water. We show that particles form in environmental waters obtained from the Pacific Ocean (seawater), natural hot springs, and soil. The particles increase in size and number in culture and produce morphologies that are highly reminiscent of living microorganisms. Our results show that these biomimetic particles consist of non-living mineral phases that incorporate trace elements and proteins, suggesting that these entities may play a role in the circulation and availability of minerals and organic molecules in the environment.

## Results

### Formation of particles in seawater

To examine whether mineral particles may spontaneously form in natural surface water, we collected seawater off the shores of Northern Taiwan and submitted the water samples to chemical analysis (see Supplementary Table S1). As expected, seawater showed high salinity (19,409‰) and electrical conductivity (EC; 38,005 μS/cm) and was slightly alkaline (pH 7.97). The main chemical elements and ions included sodium (Na), sulfate (SO_4_), calcium (Ca), and magnesium (Mg). Besides, seawater contained relatively low amounts of dissolved organic carbon (DOC; 0.547 ppm).

**Figure 1:**
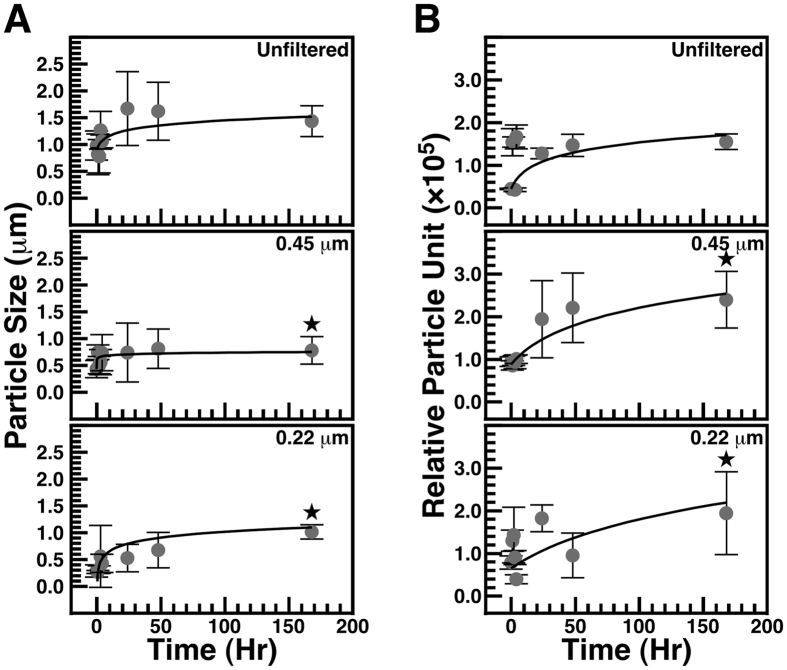
Formation of particles in unfiltered and filtered seawater. Seawater obtained off the shores of Northern Taiwan (see *Methods*) was incubated with gentle shaking at room temperature for the time indicated. Particle size (**A**) and particle number or relative particle unit (**B**) was monitored using dynamic light scattering (DLS). For comparison, seawater that had been filtered through 0.45-μm or 0.22-μm pore membranes was incubated and analyzed using DLS. Black stars indicate statistically significant results vs. time 0 (p < 0.05). See the text for more details.

We incubated the water samples at room temperature with gentle shaking for one week. In addition to seawater in its native state, we also used water filtered through either 0.45-μm or 0.22-μm pore membranes to remove particulate matter. Dynamic light scattering (DLS) analysis was used to monitor particle size and number during incubation (Fig. 1; particle numbers were expressed as relative particle units based on observations made in our previous studies^17^,^24^). While particle size and number slightly increased in unfiltered water after one week (Fig. 1A,B), the increase was not statistically significant, possibly due to the presence of particulate matter in unfiltered seawater prior to incubation. In comparison, seawater that had been filtered through either 0.45-μm or 0.22-μm pore membranes showed statistically-significant increase of particle size and number with time (Fig. 1A,B). These results suggest that seawater produces time-dependent particle formation during incubation.

### Particle-seeding potential of seawater

**Figure 2:**
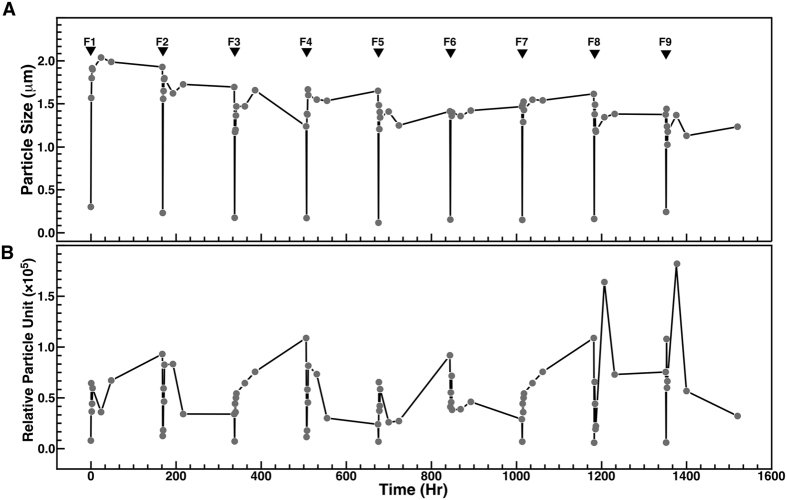
Particle-seeding potential of seawater after successive filtration-incubation cycles. Seawater was filtered through 0.45-μm pore membranes (inverted black triangles labeled F1 to F9 indicate filtration), prior to incubation at room temperature for the time indicated. Particle size (**A**) and number (**B**) was monitored using DLS. Notice the increase of particle size and number following each filtration-incubation cycle. While particles appeared to gradually decrease in size with each filtration-incubation cycle (**A**), particle number tended to slightly increase during this period (**B**).

To examine the possibility that seawater may contain a large reservoir of ions or compounds that form particles, we submitted seawater to repeated cycles of filtration through 0.45-μm pore membranes followed by incubation at room temperature to induce particle formation. After filtration, particle size and number suddenly decreased and approached zero as revealed by DLS analysis (Fig. 2A,B, F1), indicating that filtration efficiently removed the bulk of particles from the solution. Particle size rapidly increased following incubation of filtered seawater, and remained relatively stable for one week (Fig. 2A, 0–168 hrs). A second filtration (F2) again led to a sharp decrease of particle size (Fig. 2A). Notably, this cycle of filtration and particle growth could be repeated at least nine times (Fig. 2A, F1–F9), with a slight reduction in particle size being noticed with each cycle (Fig. 2A, F1–F9).

Upon incubation, particle number rapidly increased in filtered seawater (Fig. 2B, 0–168 hrs), similar to the particle number increases observed in Fig. 1B. Similar to the analysis of particle size described above (Fig. 2A), the cycle of particle number increase following successive filtration and incubation could be repeated nine times (Fig. 2B). In contrast to the changes observed for particle size, however, particle number slightly increased with each filtration-incubation cycle, reaching higher peak values after the 8^th^ and 9^th^ filtrations (Fig. 2B). These results indicate that seawater contains relatively high particle-seeding potential.

### Formation of particles in hot spring and soil waters

In order to verify that particle seeding is not limited to seawater, we also tested water obtained from hot springs in the Northern region of Taiwan. Hot spring water was collected from various locations associated with either carbonated water (i.e., Jiaoxi hot spring, Wulai hot spring) or sulfur-containing water (i.e., Yangmingshan National Park, abbreviated thereafter as YMS). Chemical analysis of hot spring water showed that Jiaoxi and Wulai water possessed comparable characteristics, whereas YMS water was more acidic (pH 2.99) and contained high DOC and sulfate levels (14,415 ppm and 410 ppm, respectively; Supplementary Table S1).

**Figure 3:**
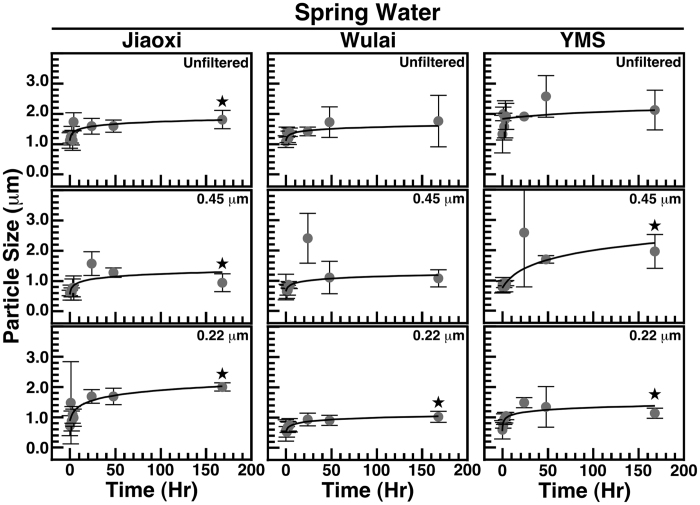
Formation of particles in natural hot spring water. Water obtained from natural hot springs located in Jiaoxi, Wulai and Yangmingshan (YMS) National Park (Northern Taiwan) was incubated at room temperature for the time indicated and particle size was monitored using DLS. In some experiments, water was filtered through 0.45-μm or 0.22-μm pore membranes as indicated, prior to incubation. Black stars indicate statistically significant results vs. time 0 (p < 0.05).

We incubated the spring water specimens at room temperature, prior to DLS analysis at regular interval (Fig. 3). Our results showed that unfilterated spring water did not produce significant particle size increase following incubation, except for Jiaoxi spring water which showed statistically significant particle size increase (Fig. 3). Following filtration, spring water from the three locations showed statistically significant particle size increases after one week, except for Wulai spring water filtered through 0.45-μm pore filter (Fig. 3).

**Figure 4:**
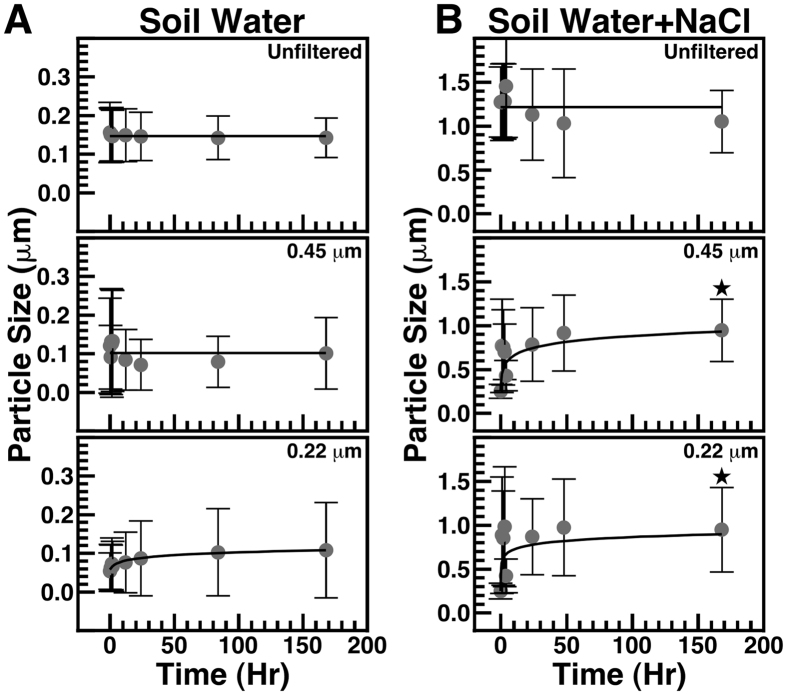
Formation of mineral particles in soil water following addition of NaCl. Soil water was prepared by mixing soil with double distilled water as described in *Methods*. The solution was centrifuged at 120000 *g* for 2 hrs at 4 °C and the supernatant was used as such (“Unfiltered”) or filtered through either 0.45-μm or 0.22-μm-pore membranes as indicated. Soil water was incubated at room temperature for the time indicated. Particle size was determined using DLS. In (**B**) NaCl (0.9%) was added to soil water prior to incubation. Black stars indicate statistically significant results vs. time 0 (p < 0.05). Notice that soil water alone did not lead to statistically significant particle formation (**A**), unless the solution was filtered and NaCl was added (**B**).

We also prepared a soil water extract in order to evaluate the particle seeding capacity of water that may percolate through soil. In this case, soil material was incubated with double distilled water, prior to centrifugation and filtration steps to remove undissolved soil material (see *Methods*). Incubation of unfiltered or filtered (0.45 μm and 0.22 μm) soil water did not produce statistically significant particle size increase after one week (Fig. 4A). This observation may be due to the presence of residual particulate matter in the prepared soil water (as the solution remained cloudy even after filtration). On the other hand, chelation of ions by organic molecules and a low level of free ions in solution may also contribute to the absence of particle formation observed in this particular soil extract. In order to evaluate this possibility, we added sodium chloride (NaCl; 0.9% w/v) to the soil water extract before incubating the mixture at room temperature for one week. After incubation, no particle size increase was noted for unfiltered soil water + NaCl; in contrast, statistically significant particle size increases were noted for both 0.45-μm and 0.2-μm filtered soil water samples in which NaCl had been added (Fig. 4B). These observations indicate that hot spring water produces particles upon incubation, whereas soil water do so only when ions are added into the solution.

### Morphology and chemical analysis of water-derived particles

**Figure 5:**
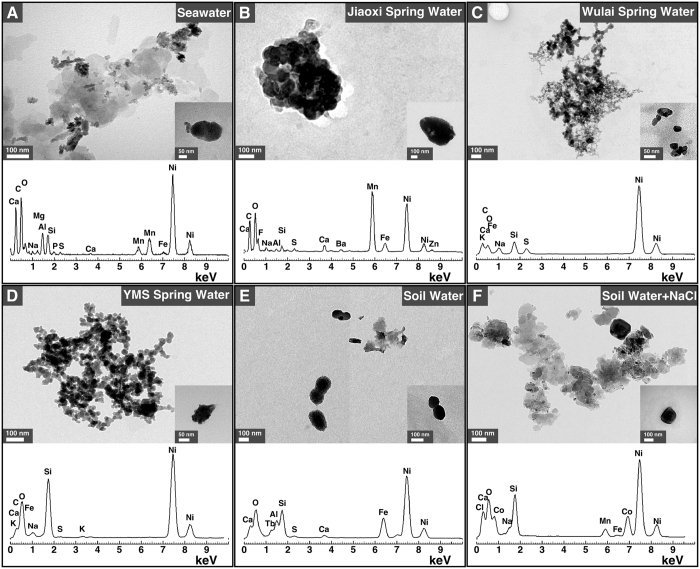
Transmission electron microscopy observations of mineral particles formed in surface waters. Seawater, hot spring water and soil water was filtered through 0.22-μm pore membranes prior to incubation at room temperature for one week as described in *Methods*. One-week old samples were centrifuged at 120000 *g* for 2 hrs at 4 °C and the resulting pellets were prepared for TEM observations without fixation or staining. EDX analysis was performed on the particles visualized under TEM. Nickel (Ni) peaks were mainly attributed to the nickel grids used as support for this analysis.

To examine the morphology and ultrastructure of the particles formed in surface water, we collected particles formed after one week of incubation and observed the samples under transmission electron microscopy (TEM) without fixation or staining (Fig. 5). The particles that formed in seawater, spring water and soil water showed round, oval or irregular morphologies with diameters ranging between 20 nm and 800 nm (Fig. 5A–F). The particles showed a round surface and tended to aggregate, resembling coccoid bacteria that aggregated to form colonies (Fig. 5A–F, insets; see also the structures resembling bacteria undergoing cellular division in Fig. 5E). Some samples formed film-like structures (Fig. 5A,C,D,F).

We used energy-dispersive X-ray spectroscopy (EDX) to identify the main chemical elements found in the particles (Fig. 5A–F, lower panels). All samples showed peaks of oxygen (O), calcium (Ca), silicon (Si), and iron (Fe) (Fig. 5A–F). Particles derived from seawater, spring water and soil water showed peaks of sulfur (S) but this element was not detected in particles derived from soil water in which NaCl had been added (Fig. 5A–E vs. F). Aluminum (Al) was noted in particles derived from seawater, Jiaoxi spring water and soil water, whereas this element was not detected in the other specimens (Fig. 5). Additional peaks of sodium (Na), magnesium (Mg), phosphorus (P), manganese (Mn), fluorine (F), barium (Ba), zinc (Zn), potassium (K), terbium (Tb), chlorine (Cl), and cobalt (Co) were also noted (Fig. 5A–F). Nickel (Ni) peaks were mainly attributed to the grids used for this analysis.

**Figure 6:**
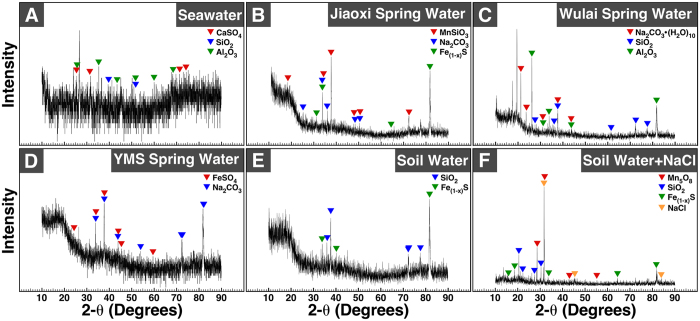
Powder X-ray diffraction analysis of mineral particles derived from surface waters. Seawater, hot spring water and soil water was filtered through 0.22-μm pore membranes prior to incubation at room temperature for one week. Particles found in one-week old samples were centrifuged at 120000 *g* for 2 hrs at 4 °C and the resulting pellets were prepared for X-ray diffraction analysis as described in *Methods*.

Powder X-ray diffraction (XRD) analysis was used to identify the chemical formulas of the minerals found in the particles (Fig. 6). Each particle sample produced distinct diffraction peaks suggesting the presence of crystalline materials (Fig. 6A–F). Comparison of these peaks with existing XRD spectrum database indicated that each particle sample contained a mixture of two to four crystals (Fig. 6A–F, chemical formulas are shown in the top right corner along with the peaks corresponding to each mineral). Seawater-derived particles contained mineral phases of calcium sulfate (CaSO_4_), silicon oxide (SiO_2_), and aluminum oxide (Al_2_O_3_) (Fig. 6A). Jiaoxi and Wulai spring waters produced mineral particles containing sodium carbonate (Na_2_CO_3_), while YMS spring water yielded particles of iron (II) sulfate (FeSO_4_) (Fig. 6B–D), consistent with the descriptions of these hot springs as carbonated and sulfur hot springs, respectively. Other minerals were also noted in particles derived from hot spring water, including pyroxmangite (MnSiO_3_), iron sulfide (Fe_(X–1)_S), silicon oxide, and aluminum oxide (Fig. 6). Mineral particles derived from soil water mainly contained silicon oxide and iron sulfide (Fig. 6E). Particles produced in soil water in which NaCl was added showed peaks corresponding to manganese oxide (Mn_5_O_8_), silicon oxide, iron sulfide and sodium chloride (Fig. 6F), indicating that at least part of the particles detected in this sample represented NaCl. Overall, the particles had a low level of crystallinity as shown by the small number and low intensity of diffraction peaks (Fig. 6). While minor variations were noted, the XRD results obtained were consistent with the EDX results described above (Fig. 5) and the initial chemical analysis of the water samples (Supplementary Table S1).

### Proteomic analysis of mineralo-organic particles derived from surface water

The relatively rounded surface of the mineral particles (Fig. 5) and their apparently low crystallinity (Fig. 6) suggested that the minerals may contain molecules that inhibit mineralization and contribute to the formation of amorphous phases. In order to examine this possibility, we performed a proteomic analysis of the particles using a methodology that we established earlier to study the protein composition of mineralo-organic particles derived from human body fluids^16^. We observed earlier that mineralo-organic particles derived from human body fluids bind to a wide range of proteins, including coagulation factors, calcification inhibitors, complement proteins, protease inhibitors, and lipid carriers^16^.

Using the same liquid chromatography-tandem mass spectrometry (LC/MS-MS) approach, we observed that mineral particles derived from surface water contained proteins belonging to the Bacteria, Plantae and Animalia kingdoms (Supplementary Table S2). Mineral particles formed in soil water in which NaCl had been added harbored by far the largest amount and diversity of proteins (Supplementary Table S2). These observations indicate that mineral particles that form in surface water may bind to organic molecules present in solution, forming mineralo-organic complexes.

### Biomimetic morphologies of particles derived from soil water

**Figure 7:**
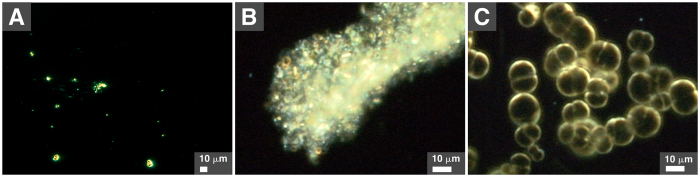
Biomimetic morphologies of mineral particles formed in soil water. Soil water was prepared by mixing one volume of soil with two volumes of double distilled water. The mixture was autoclaved, followed by centrifugation at 1800 *g* for 30 min. The supernatant was filtered through a 0.2-μm pore filter and frozen at −20 °C for seven days. The resulting solution was thawed at room temperature before observation by dark-field optical microscopy without fixation or staining. Various morphologies were observed, including small, dispersed particles (**A**), aggregated particles (**B**), and large formations reminiscent of living microorganisms undergoing cellular division (**C**).

We observed earlier that mineral particles that precipitate in the presence of proteins form round particles that are highly reminiscent of living microorganisms^10^,^11^,^18^,^22^. We examined the morphologies of soil-water-derived particles using dark-field optical microscopy without fixation or staining, a technique that allows us to follow the formation of the mineral particles during incubation. We observed that soil water produced particles of various morphologies, including small round particles (Fig. 7A) that tended to aggregate to form clumps (Fig. 7B). Notably, large, round particles that formed cell-like structures were observed in soil water samples (Fig. 7C). These biomimetic particles formed relatively large structures that were highly similar to cells undergoing division (Fig. 7C).

## Discussion

While synthetic nanomaterials have been studied for their possible technological and biomedical applications, less information is available regarding the formation and biomimetic properties of natural NPs in the environment. We describe here that biomimetic mineralo-organic particles form in surface water obtained from the ocean, hot springs, and soil. These entities consist of mineralo-organic particles of mixed crystalline compositions containing proteins belonging mainly to bacteria but also to plants and animals. The biomimetic mineralo-organic particles are highly similar to the NPs that we detected earlier in human body fluids, and which we referred as bions^20^,^22^, suggesting that such mineralo-organic particles may not only form in the human body but also in environmental surface water. Similar mineral particles were also described in human body fluids by other authors including Price^38^,^39^, Jahnen-Dechent^37^,^40^, Evan^41^,^42^, Smith^43^,^44^, and others (see also ref. 22 and references therein). The observations presented here indicate that, in addition to the carbonate-, sulfate- and phosphate-based minerals identified earlier^10^,^11^,^18^,^20^,^23^,^24^, mineralo-organic particles may incorporate other minerals (oxides, silicates, and sulfides) that reflect the initial composition of the milieu in which the particles formed. By binding to trace elements and organic molecules in environmental waters, these mineralo-organic particles may be involved in the circulation and availability of trace elements and organics in the environment.

We observed that seawater possesses a large particle-formation potential (Fig. 2), possibly due to high levels of dissolved ions such as sodium, calcium and magnesium (Supplementary Table S1). Mineralo-organic particles that formed in soil water in which NaCl had been added yielded the largest amount of proteins in our experiments (Supplementary Table S2), an observation which may be due to a larger amount of particles produced under these conditions. Our previous observations that various organic molecules bind to bions formed in body fluids^11^,^12^,^13^,^16^,^20^,^22^,^26^ suggest that additional organic compounds such as humic matter, peptidoglycans and polysaccharides may also be present in the particles described here. Notably, such organic molecules appear to play a role in the formation of morphologies similar to living microorganisms as reported here (Figs 5 and 7).

Mineral particles with morphologies reminiscent of living microorganisms have been described earlier in previous studies. For instance, small mineralized entities called nannobacteria (spelled with two N’s in this context) have been described in various natural environments, including the carbonate hot springs of Viterbo in Italy^45^, chalcocite (Cu_2_S) deposits in Northern Chile^46^, and fresh-water streams and water pipes in the region of Texas^47^. These nannobacteria have been interpreted as living or fossilized forms of bacteria that may participate in the deposition of minerals in nature. On the other hand, the observations described here and in previous studies^14^,^18^,^22^ indicate that mineralo-organic particles may mimic living microorganisms in several ways. For instance, we show here that mineralo-organic particles can increase in size and number during incubation (Figs 1, 2, 3, 4); they have biomimetic morphologies (Figs 5 and 7); and they bind to organic molecules such as proteins derived from the starting water (Supplementary Table S2). Our observations therefore reinforce the concept that morphology is clearly insufficient to prove existence of living organisms or fossils^14^,^18^. Our conclusion is also in agreement with previous reports of nanobacteria-like particles in the Tataouine meteorite^48^ and in water derived from hot springs in China^49^, findings which were attributed to the precipitation of amorphous calcium carbonate.

Our study shares some analogies with the seminal work of Chin *et al*. who showed that dissolved organic matter (DOM) found in marine seawater spontaneously assembles to form a polymer gel containing calcium carbonate^50^. In the latter work, ocean water that had been filtrated through 0.22-μm pore membrane produced colloid particles with diameters of 2 to 200 nm upon incubation at 20 °C for several hours. These particles gradually increased in size (200 nm to 1 μm), leading to the formation of a network of organic matter containing carbohydrates, proteins, and lipids found in seawater. Calcium carbonate crystals also formed inside the network and this process was attributed to increased pH and a Donnan effect which suggests that the level of calcium ions within the organic network should be higher than outside due to the polyanionic nature of the network^50^. A similar transition from DOM to particulate organic matter (POM) has been described in various water environments and may represent a general phenomenon with widespread repercussions for geochemical cycles, nutrient availability and prey-predator interactions in oceans^51^. Further studies are needed to examine the possible relationship between this polymer gel and the formation of mineralo-organic particles described in the present study.

From another perspective, Reich described round particles, which he also termed “bions” (although there is no direct connection with our use of the term; see also our previous work^20^), in decaying organic matter and soil-water specimens^52^. These particles were interpreted by Reich as primitive life-forms or pro-life-forms that may form from organic matter. Separately, Grad^53^, Snyder^54^ and DeMeo^55^ observed similar structures that they respectively described as “primordial forms”, “cell-like structures”, or “protocellular forms” in aqueous extracts of soil prepared in a manner similar to the one we used to prepare soil water extract in the present study. Notably, the particles observed by these authors are strikingly similar to the non-living mineralo-organic particles described here (Figs 5 and 7) and in previous studies^11^,^18^,^19^,^20^,^24^. We believe that our findings provide a more rational explanation for the descriptions of primitive life-forms made by Reich and other authors. Accordingly, these non-living mineral-organic particles have been shown to spontaneously form in solutions containing abundant ions and organic molecules^10^,^11^,^12^,^13^,^14^,^15^,^16^,^17^,^18^,^19^,^20^,^21^,^22^,^23^,^24^,^25^,^26^.

Our results suggest that mineralo-organic particles form in environmental water obtained from various sources. While the particles described here are highly similar to the descriptions of nanobacteria and nannobacteria reported in previous studies, our results indicate that these particles are non-living entities that mimic living microorganisms in several ways. As such, the mineralo-organic particles increase in size and number in culture; they harbor cell-like morphologies; and they bind to organic molecules such as proteins. We speculate that these mineralo-organic particles may affect the circulation and availability of trace elements and organics in nature. The approach developed in the present study provides a platform to examine this possibility and study in more details the possible roles and fate of mineralo-organic particles in the environment.

## Methods

### Water samples

Water samples were collected between April 22, 2008 and May 25, 2012. Seawater was collected off the shores of Northern Taiwan. Hot spring water was collected from Jiaoxi, Wulai and YMS hot springs in Taiwan. Global positioning system (GPS) coordinates of the sampling sites are listed in Supplementary Table S1. Water samples were transported in clean, sterile bottles. Wulai hot spring water contained small amounts of white solid material that sedimented at the bottom of the bottles with time. Unless indicated otherwise, water samples were filtered through either 0.45-μm or 0.22-μm-pore membranes (Millipore) before use.

To prepare soil water, one volume of soil collected on the campus of Chang Gung University (see Supplementary Table S1 for GPS coordinates) was mixed with two volumes of double distilled water. The mixture was vigorously mixed for 1 hr at room temperature, prior to centrifugation at 1800 *g* for 30 min. The supernatant was collected and filtered through two layers of filter paper (Whatman). The solution was successively filtered through 0.45-μm or 0.22-μm-pore membranes. Samples were stored at 4 °C before use. In some experiments, NaCl (0.9%) was added to the mixture before mixing and centrifugation as above. The experiments shown in Fig. 7 were performed based on a protocol similar to the one used in previous studies^52^,^53^,^54^,^55^. Briefly, in these experiments, the soil-water mixture was autoclaved, followed by centrifugation at 1800 *g* for 30 min. The supernatant was filtered through a 0.2-μm pore membrane and frozen at −20 °C for seven days. The solution was thawed at room temperature before observation by dark-field optical microscopy as described below.

### Chemical analysis

Water pH, salinity and electrical conductivity (EC) were determined using the 556 MPS multi-parameter instrument (YSI). Dissolved organic carbon (DOC), dissolved ions and trace elements were determined by the Amia Company using inductively coupled plasma-optical emission spectrometry (ICP-OES; Varian 710–ES).

### Water treatment

Unfiltered and filtered water samples were incubated at room temperature for one week on a RS-101 rocking shaker (Firstek). In Fig. 2, incubated seawater was filtered through 0.45-μm membranes before re-incubation, and the filtration-incubation cycle was performed nine times.

### Dynamic light scattering

Particle sizing and counting was performed as described previously^19^ using a Coulter N4 Plus Submicron Particle Size Analyzer (Beckman Coulter). In brief, incubated water (1 ml) was transferred to disposable plastic cuvettes (Kartell), prior to gentle mixing by inversion. Reading was performed at an incident angle of 90° at room temperature. Relative particle units (or particle number) correlated in a linear manner with the number of particles observed under optical dark-field microscopy and TEM^17^.

### Electron microscopy

Water samples were centrifuged at 120000 *g* for 2 hrs at 4 °C to pellet mineral particles. Pellets were washed in HEPES buffer (20 mM HEPES, 140 mM NaCl, pH 7.4) using the same centrifugation step, prior to resuspension in 100% ethanol. Solutions were sonicated 25 min on ice. Aliquots were deposited onto nickel grids coated with a Formvar-carbon film, prior to drying in air under yellow fluorescent light. Dried grids were observed without fixation or staining under a JEOL 2100 transmission electron microscope operated at 200 kV.

### Energy-dispersive X-ray spectroscopy

Mineral particles were prepared as above for electron microscopy. EDX spectra were obtained in triplicate with the INCA Energy Microanalysis System (Oxford Instruments). Representative EDX spectra are shown in Fig. 5.

### Powder X-ray diffraction analysis

Mineral particles were prepared as above for electron microscopy. Washed particle pellets were dried at 60 °C. Dried pellets were analyzed using the X’PertPRO diffractometer (PANalytical). Chemical formulas were obtained by comparing experimental spectra with the database of the Materials Data Incorporated (MDI)’s Jade and X’Pert High Score (PANalytical) data analysis software.

### Proteomic analysis

Water samples were centrifuged at 120000*g* for 2 hrs at 4 °C to pellet mineral particles. Pellets were washed in HEPES buffer, prior to freeze-drying using a CentriVap Benchtop Vacuum Concentrator (Labconco). Proteomic analysis was performed as before^16^. Pellets were treated with a solution of 25 mM ammonium bicarbonate containing 200 mM of the protein-reducing reagent dithiotreitol and incubated for 30 min at 65 °C. Pellets were treated with 25 mM ammonium bicarbonate containing 200 mM of the protein-alkylating reagent iodothiotreitol for 30 min at room temperature in the dark. In-solution trypsin digestion was performed using sequencing-grade porcine trypin (Promega; 20 μg/ml) at 37 °C overnight (approximately 1 volume of trypsin was used for 25 volumes of proteins). The resulting peptides were desalted in a homemade column, dried in vacuum centrifuge, and loaded onto a Zorbax 300SB-C18 reverse-phase liquid chromatography column (Agilent Technologies). Peptide separation and elution as well as data analysis was performed as described^16^. MS data files were analyzed using the Proteome Discoverer Software (version 1.3.0.339; Thermo Fisher). All samples were compared individually against bacterial, plant, and mammalian taxonomies of the Swiss-Prot database using the MASCOT software (version 2.2; Matrix Science). The proteins identified had at least one identified unique peptide. Proteins interpreted as contaminants were discarded (e.g., keratins, caseins, beta-lactoglobulin).

### Optical dark-field microscopy

Washed particles resuspended in double distilled water were deposited on microscopy glass slides. Samples were observed without fixation or staining using a BX-51 optical microscope (Olympus) equipped with a dark-field condenser (Cerbe Distribution) and a 100 × oil immersion UPlanFLN objective with iris (Olympus). Images were acquired with a Spot Flex color, charge-coupled device camera (Diagnostic Instruments).

### Statistical analysis

Experiments were performed at least three times. Values are expressed as means ± standard deviation. Statistical analysis was performed using Student’s *t* test. A statistical significance threshold of p < 0.05 was used.

## Additional Information

**How to cite this article**: Wu, C.-Y. *et al*. Formation and characteristics of biomimetic mineralo-organic particles in natural surface water. *Sci. Rep.* **6**, 28817; doi: 10.1038/srep28817 (2016).

## Supplementary Material

Supplementary Information
