# Supplementary material for: Formation and characteristics of biomimetic mineralo-organic particles in natural surface water
Source: Sci Rep. 2016 Jun 28;6:28817. doi: 10.1038/srep28817 (PMC4923871; doi:10.1038/srep28817)
Supplement: Supplementary Information [file srep28817-s1.doc]

**Supplementary Information**

**Formation and characteristics of biomimetic mineralo-organic particles in natural surface water**

Cheng-Yeu Wu^1,2,3^, Jan Martel^1,2^, Tsui-Yin Wong^1,2,4^, David Young^1,5^, Chien-Chun Liu^6^, Cheng-Wei Lin^1,2^, and John D. Young^1,2,7,8^

^1^ Laboratory of Nanomaterials, Chang Gung University, Taoyuan, Taiwan, Republic of China

^2^ Center for Molecular and Clinical Immunology, Chang Gung University, Taoyuan, Taiwan, Republic of China

^3^ Research Center of Bacterial Pathogenesis, Chang Gung University, Taoyuan, Taiwan, Republic of China

^4^ Center for General Education, Chang Gung University of Science and Technology,

Chiayi, Taiwan, Republic of China

^5^ Department of Materials Science and Engineering, Massachusetts Institute of Technology, Cambridge, Massachusetts, United States of America

^6^ Graduate Institute of Biomedical Sciences, College of Medicine, Chang Gung University, Taoyuan, Taiwan, Republic of China

^7^ Biochemical Engineering Research Center, Ming Chi University of Technology, New Taipei City, Taiwan, Republic of China

^8^ Laboratory of Cellular Physiology and Immunology, The Rockefeller University, New York, New York, United States of America

**Supplementary Table S1. Chemical analysis of surface water samples used in the present study**

| **Sample** | **Seawater** | **Spring Water** | | | **Soil Water** |
| --- | --- | --- | --- | --- | --- |
|  |  | **Jiaoxi** | **Wulai** | **YMS** |  |
| **GPS Location** | 24.89°N, 121.85°W | 24.82°N, 121.77°W | 24.86°N, 121.55°W | 25.14°N, 121.52°E | 25.03°N, 121.38°E |
| **Sampling Date** | April 22, 2008 | May 14, 2008 | May 25, 2008 | April 22, 2008 | May 25, 2012 |
| pH | 7.97 | 7.82 | 7.12 | 2.99 | 7.23 |
| Salinity (‰) | 19,409 | 20 | 18 | 38 | 4 |
| EC (μS/cm) | 38,005 | 696 | 619 | 1,125 | 178 |
| DOC (ppm) | 0.547 | 1.520 | 1.436 | 14.415 | 3.206 |
| SO_4_ (ppm) | 1,592 | ND | 212 | 410 | 10 |
| TP (ppm) | 0.004 | 0.006 | 0.009 | 0.012 | 0.024 |
| Na (ppm) | 13,923 | 188 | 40 | 48 | 15 |
| Ca (ppm) | 389.9 | 11.2 | 74.4 | 88.9 | 24.6 |
| Mg (ppm) | 52.6 | 2.8 | 35.9 | 39.1 | 2.8 |
| Si (ppm) | 1.5 | 19.4 | 60.7 | 62.4 | 23.2 |
| Al (ppm) | 0.23 | ND | 0.08 | 1.08 | 5.51 |
| Co (ppm) | ND | ND | ND | ND | 0.0051 |
| Cd (ppm) | 0.0015 | 0.0008 | ND | ND | ND |
| Cr (ppm) | 0.0031 | ND | 0.0009 | 0.0024 | ND |
| Cu (ppm) | ND | ND | ND | ND | 0.0064 |
| Mn (ppm) | 0.011 | 0.017 | 0.6 | 0.81 | 0.022 |
| Ni (ppm) | 0.0077 | 0.0313 | 0.0007 | 0.0012 | 0.0036 |
| Pb (ppm) | 0.0108 | ND | ND | 0.0014 | ND |
| Sn (ppm) | 0.0216 | ND | 0.0204 | 0.0058 | 0.0152 |
| Fe (ppm) | 0.0043 | ND | 0.022 | 0.0633 | 4.415 |
| Zn (ppm) | 0.0136 | 0.0124 | 0.0081 | 0.0042 | 0.026 |

EC: electrical conductivity; DOC: dissolved organic carbon; GPS: global positioning system; ND: not detected; TP: total phosphate; YMS: Yangmingshan.

| **Supplementary Table S2. Proteomics analysis of mineralo-organic particles derived from surface water** | | | | | |  |  |  |  |  |  |  |  |  |  |  |  |  |  |  |  |  |  |  |  |  |  |  |
| --- | --- | --- | --- | --- | --- | --- | --- | --- | --- | --- | --- | --- | --- | --- | --- | --- | --- | --- | --- | --- | --- | --- | --- | --- | --- | --- | --- | --- |
| **No.** | **Kingdom** | **Protein identified** | **UniProt** | **MW** | **Seawater** | |  |  | **Jiaoxi-SW^a^** | | |  | **Wulai-SW** | | |  | **YMS-SW** | |  |  | **Soil-Water** | |  |  | **Soil-Water + NaCl** | | | |
|  |  |  | **Accession No.** | **(kDa)** | **C^b^** | **P^c^** | **UP^d^** | **PSM^e^** | **C** | **P** | **UP** | **PSM** | **C** | **P** | **UP** | **PSM** | **C** | **P** | **UP** | **PSM** | **C** | **P** | **UP** | **PSM** | **C** | **P** | **UP** | **PSM** |
| 1 | Bacteria | ATP synthase subunit beta | A1SHJ1 | 52.6 | – | – | – | – | – | – | – | – | – | – | – | – | – | – | – | – | – | – | – | – | 15.08 | 5 | 4 | 9 |
| 2 |  | 50S ribosomal protein L7/L12 | A0LRL2 | 13.5 | – | – | – | – | – | – | – | – | – | – | – | – | – | – | – | – | – | – | – | – | 18.46 | 2 | 2 | 2 |
| 3 |  | ATP synthase subunit beta | Q4L7Y4 | 51.5 | – | – | – | – | – | – | – | – | – | – | – | – | – | – | – | – | – | – | – | – | 5.74 | 2 | 1 | 4 |
| 4 |  | DNA-directed RNA polymerase subunit alpha | Q0RRP3 | 37.8 | – | – | – | – | – | – | – | – | – | – | – | – | – | – | – | – | – | – | – | – | 6.88 | 2 | 1 | 2 |
| 5 |  | DNA-directed RNA polymerase subunit alpha | Q82QR5 | 36.5 | – | – | – | – | – | – | – | – | – | – | – | – | – | – | – | – | – | – | – | – | 6.51 | 2 | 1 | 2 |
| 6 |  | 10 kDa chaperonin | A1SMW2 | 10.4 | – | – | – | – | – | – | – | – | – | – | – | – | – | – | – | – | – | – | – | – | 15.46 | 1 | 1 | 2 |
| 7 |  | 30S ribosomal protein S8 | A0QKZ8 | 14.4 | – | – | – | – | – | – | – | – | – | – | – | – | – | – | – | – | – | – | – | – | 15.15 | 1 | 1 | 1 |
| 8 |  | Major carboxysome shell protein 1C | P45688 | 9.9 | – | – | – | – | – | – | – | – | – | – | – | – | 11.22 | 1 | 1 | 1 | – | – | – | – | – | – | – | – |
| 9 |  | Single-stranded DNA-binding protein | P0A611 | 17.3 | – | – | – | – | – | – | – | – | – | – | – | – | – | – | – | – | – | – | – | – | 10.98 | 1 | 1 | 1 |
| 10 |  | 50S ribosomal protein L7/L12 | A1SEI9 | 13.4 | – | – | – | – | – | – | – | – | – | – | – | – | – | – | – | – | – | – | – | – | 9.92 | 1 | 1 | 1 |
| 11 |  | Pyridoxal biosynthesis lyase PdxS | Q2LXR2 | 31.6 | – | – | – | – | – | – | – | – | – | – | – | – | – | – | – | – | – | – | – | – | 5.12 | 1 | 1 | 2 |
| 12 |  | Elongation factor G (Fragment) | P29541 | 37.3 | – | – | – | – | – | – | – | – | – | – | – | – | – | – | – | – | – | – | – | – | 4.69 | 1 | 1 | 1 |
| 13 |  | Peptide chain release factor 2 | B8HBH8 | 41.1 | – | – | – | – | – | – | – | – | – | – | – | – | – | – | – | – | – | – | – | – | 4.58 | 1 | 1 | 1 |
| 14 |  | Enolase | A0JU21 | 45.0 | – | – | – | – | – | – | – | – | – | – | – | – | – | – | – | – | – | – | – | – | 4.46 | 1 | 1 | 1 |
| 15 |  | Elongation factor Tu | A7GK18 | 42.9 | – | – | – | – | – | – | – | – | – | – | – | – | – | – | – | – | – | – | – | – | 4.3 | 1 | 1 | 1 |
| 16 |  | Ketol-acid reductoisomerase | A9WP08 | 36.9 | – | – | – | – | – | – | – | – | – | – | – | – | – | – | – | – | – | – | – | – | 3.81 | 1 | 1 | 2 |
| 17 |  | 60 kDa chaperonin | A5CTX3 | 56.7 | – | – | – | – | – | – | – | – | – | – | – | – | – | – | – | – | – | – | – | – | 3.71 | 1 | 1 | 2 |
| 18 |  | Elongation factor Tu | A0LRL8 | 44.0 | – | – | – | – | – | – | – | – | – | – | – | – | – | – | – | – | – | – | – | – | 3.53 | 1 | 1 | 2 |
| 19 |  | Cell division protein FtsZ | P45500 | 41.1 | – | – | – | – | – | – | – | – | – | – | – | – | – | – | – | – | – | – | – | – | 3.51 | 1 | 1 | 1 |
| 20 |  | General secretion pathway protein N | P29040 | 27.5 | 3.45 | 1 | 1 | 1 | – | – | – | – | – | – | – | – | – | – | – | – | – | – | – | – | – | – | – | – |
| 21 |  | Glyceraldehyde-3-phosphate dehydrogenase | A0QWW2 | 35.9 | – | – | – | – | – | – | – | – | – | – | – | – | – | – | – | – | – | – | – | – | 2.94 | 1 | 1 | 1 |
| 22 |  | EPTC-inducible aldehyde dehydrogenase | P46369 | 55.0 | – | – | – | – | – | – | – | – | – | – | – | – | – | – | – | – | – | – | – | – | 2.77 | 1 | 1 | 1 |
| 23 |  | ATP synthase subunit alpha | A0Q2Z6 | 55.2 | – | – | – | – | – | – | – | – | – | – | – | – | – | – | – | – | – | – | – | – | 2.59 | 1 | 1 | 2 |
| 24 |  | Elongation factor Tu | A1T056 | 43.2 | – | – | – | – | – | – | – | – | – | – | – | – | – | – | – | – | – | – | – | – | 2.54 | 1 | 1 | 2 |
| 25 |  | Chaperone protein DnaK | C4K3I6 | 68.9 | – | – | – | – | – | – | – | – | – | – | – | – | – | – | – | – | – | – | – | – | 2.52 | 1 | 1 | 2 |
| 26 |  | 60 kDa chaperonin | B7J561 | 58.6 | – | – | – | – | – | – | – | – | – | – | – | – | 2.36 | 1 | 1 | 1 | – | – | – | – | – | – | – | – |
| 27 |  | Isocitrate dehydrogenase [NADP] | Q8RQL9 | 79.2 | – | – | – | – | – | – | – | – | – | – | – | – | – | – | – | – | – | – | – | – | 2.33 | 1 | 1 | 2 |
| 28 |  | Polyribonucleotide nucleotidyltransferase | A6W815 | 79.0 | – | – | – | – | – | – | – | – | – | – | – | – | – | – | – | – | – | – | – | – | 2.28 | 1 | 1 | 2 |
| 29 |  | ATP synthase subunit alpha | Q0RDB2 | 59.5 | – | – | – | – | – | – | – | – | – | – | – | – | – | – | – | – | – | – | – | – | 2.17 | 1 | 1 | 1 |
| 30 |  | Polyribonucleotide nucleotidyltransferase | A0JUV8 | 80.0 | – | – | – | – | – | – | – | – | – | – | – | – | – | – | – | – | – | – | – | – | 2.01 | 1 | 1 | 1 |
| 31 |  | Chaperone protein DnaK | P64410 | 65.4 | – | – | – | – | – | – | – | – | – | – | – | – | – | – | – | – | – | – | – | – | 1.99 | 1 | 1 | 1 |
| 32 |  | Polyribonucleotide nucleotidyltransferase | A4X4P8 | 83.9 | – | – | – | – | – | – | – | – | – | – | – | – | – | – | – | – | – | – | – | – | 1.91 | 1 | 1 | 1 |
| 33 |  | DNA-directed RNA polymerase subunit beta 1 | Q5YQP4 | 127.8 | – | – | – | – | – | – | – | – | – | – | – | – | – | – | – | – | – | – | – | – | 1.38 | 1 | 1 | 1 |
| 34 |  | Multifunctional 2-oxoglutarate metabolism enzyme | A0R2B1 | 135.9 | – | – | – | – | – | – | – | – | – | – | – | – | – | – | – | – | – | – | – | – | 1.14 | 1 | 1 | 2 |
| 35 |  | Aconitate hydratase | P37032 | 98.1 | – | – | – | – | – | – | – | – | – | – | – | – | – | – | – | – | – | – | – | – | 1.12 | 1 | 1 | 1 |
| 36 |  | DNA-directed RNA polymerase subunit beta' | Q6A6K7 | 143.4 | – | – | – | – | – | – | – | – | – | – | – | – | – | – | – | – | – | – | – | – | 0.93 | 1 | 1 | 1 |
| 37 | Plantae | Unknown protein 1 (Fragment) | P86104 | 1.4 | 100 | 2 | 2 | 3 | – | – |  | – | 100 | 2 | 2 | 3 | 100 | 1 | 1 | 2 | 91.67 | 1 | 1 | 1 | 100 | 1 | 1 | 2 |
| 38 |  | Putative cytochrome c oxidase subunit II PS17 (Fragments) | P84733 | 1.7 | – | – | – | – | – | – |  | – | – | – | – | – | – | – | – | – | 5 | 1 | 1 | 1 | – | – | – | – |
| 39 |  | ATP synthase subunit beta, chloroplastic | O03063 | 31.0 | – | – | – | – | – | – |  | – | – | – | – | – | – | – | – | – | – | – | – | – | 4.58 | 1 | 1 | 2 |
| 40 |  | ADP, ATP carrier protein | P27080 | 33.5 | – | – | – | – | – | – |  | – | – | – | – | – | – | – | – | – | – | – | – | – | 3.9 | 1 | 1 | 1 |
| 41 |  | ATP synthase subunit alpha, chloroplastic | P41602 | 53.3 | – | – | – | – | – | – |  | – | – | – | – | – | – | – | – | – | – | – | – | – | 2.63 | 1 | 1 | 2 |
| 42 |  | Heat shock 70 kDa protein 18 | Q9C7X7 | 68.3 | – | – | – | – | – | – |  | – | – | – | – | – | – | – | – | – | – | – | – | – | 2.59 | 1 | 1 | 2 |
| 43 | Animalia | ATP synthase subunit beta, mitochondrial | P00829 | 56.2 | – | – | – | – | – | – | – | – | 2.08 | 1 | 1 | 2 | – | – | – | – | – | – | – | – | 4.73 | 2 | 2 | 3 |
| 44 |  | Heat shock-related 70 kDa protein 2 | P86204 | 21.3 | – | – | – | – | – | – | – | – | – | – | – | – | – | – | – | – | – | – | – | – | 8.33 | 1 | 1 | 2 |
| 45 |  | Malectin | Q5FVQ4 | 32.4 | – | – | – | – | – | – | – | – | – | – | – | – | – | – | – | – | – | – | – | – | 3.78 | 1 | 1 | 1 |
| 46 |  | ATP synthase subunit alpha, mitochondrial | P15999 | 59.7 | – | – | – | – | – | – | – | – | – | – | – | – | – | – | – | – | – | – | – | – | 2.35 | 1 | 1 | 2 |
| 47 |  | Xanthine dehydrogenase/oxidase | P80457 | 146.7 | – | – | – | – | – | – | – | – | – | – | – | – | – | – | – | – | – | – | – | – | 0.90 | 1 | 1 | 2 |
| 48 |  | DNA ligase 3 | P49916 | 112.8 | – | – | – | – | – | – | – | – | – | – | – | – | – | – | – | – | – | – | – | – | 0.89 | 1 | 1 | 1 |
| 49 |  | Collagen alpha-1(III) chain | P02461 | 138.5 | – | – | – | – | – | – | – | – | 0.82 | 1 | 1 | 1 | – | – | – | – | – | – | – | – | – | – | – | – |
| 50 |  | Ankyrin repeat and KH domain-containing protein 1 | Q8IWZ3 | 269.3 | – | – | – | – | – | – | – | – | – | – | – | – | – | – | – | – | – | – | – | – | 0.28 | 1 | 1 | 1 |
| ^a^ Spring water (SW). | | | | | | | | |  |  |  |  |  |  |  |  |  |  |  |  |  |  |  |  |  |  |  |  |
| ^b^ Coverage (C): percentage of protein sequence covered by the identified peptides. | | |  |  |  |  |  |  |  |  |  |  |  |  |  |  |  |  |  |  |  |  |  |  |  |  |  |  |
| ^c^ Peptide (P): number of peptides identified in each protein. | | |  |  |  |  |  |  |  |  |  |  |  |  |  |  |  |  |  |  |  |  |  |  |  |  |  |  |
| ^d^ Unique Peptide (UP): number of unique peptides identified for each protein. | | |  |  |  |  |  |  |  |  |  |  |  |  |  |  |  |  |  |  |  |  |  |  |  |  |  |  |
| ^e^ Peptide spectrum match (PSM): total number of peptide spectrum match for each protein, including redundant match. | | | | | | |  |  |  |  |  |  |  |  |  |  |  |  |  |  |  |  |  |  |  |  |  |  |
